# Is anti-platelet therapy interruption a real clinical issue? Its implications in dentistry and particularly in periodontics

**DOI:** 10.4103/0972-124X.60223

**Published:** 2009

**Authors:** A. Jaya Kumar, M. Meena Kumari, Nupur Arora, A. Haritha

**Affiliations:** 1 *Department of Periodontics, Sri Sai College of Dental Surgery, Vikarabad, Andhra Pradesh, India*; 2 *Department of Periodontics, Sri Sai College of Dental Surgery, Vikarabad, Andhra Pradesh, India*; 3 *Department of Periodontics, Sri Sai College of Dental Surgery, Vikarabad, Andhra Pradesh, India*; 4 *Department of Periodontics, Sri Sai College of Dental Surgery, Vikarabad, Andhra Pradesh, India*

**Keywords:** Anti-platelet therapy, dental and periodontal treatment, decision-making

## Abstract

The use of anti-platelet therapy has reduced the mortality and morbidity of cardiovascular disease remarkably. A considerable number of patients presenting before a dentist or periodontist give a history of anti-platelet therapy. A clinical dilemma whether to discontinue the anti-platelet therapy or continue the same always confronts the practitioner. Diverse opinions exist regarding the management of such patients. While one group of researchers advise continuation of anti-platelet therapy rather than invite remote, but possible, thromboembolic events, another group encourages discontinuation for variable periods. This study aims at reviewing the current rationale of anti-platelet therapy and the various options available to a clinician, with regard to the management of a patient under anti-platelet therapy. Current recommendations and consensus favour no discontinuation of anti-platelet therapy. This recommendation, however, comes with a rider to use caution and consider other mitigating factors as well. With a large number of patients giving a history of anti-platelet therapy, the topic is of interest and helps a clinician to arrive at a decision.

## INTRODUCTION

Cardiovascular diseases account for the highest mortality and morbidity worldwide. With increasing awareness, health consciousness, and preventive and pre-emptive strategies, there is a striking decrease of cardiovascular mortality, resulting in fewer deaths compared to the earlier decades. The introduction of preventive and maintenance anti-platelet therapy has to a certain extent contributed to this decline.

Anti-platelet treatment has been reported to have reduced the overall mortality of vascular disease by 15% and non-fatal vascular complications by 30%.[1]

With millions of health conscious people using anti-platelet drugs, dental practitioners are frequently confronted with clinical situations where a decision has to be made about patient management, in view of the medical history.

Till the very recent past, the recommendation was to stop anti-platelet therapy, to avoid excessive postoperative bleeding. The stoppage of anti-platelet therapy was recommended for a variable period, that is, from three to seven days before the planned event. There was a total lack of concern or underestimation of the thromboembolic events compared to hemorrhagic risks.

There has been a reawakening of interest in this hitherto underestimated risk. To understand the rationale of the whole phenomenon, a brief recapitulation of the basic issues need to be reviewed.

## PHYSIOLOGY OF PLATELETS AND ITS FUNCTION

Platelets are small disk-shaped cells without a nucleus, derived from bone marrow, and when released into the blood have a life span of 10 days. Platelets circulate in vessels and do not show any tendency toward adhesion to the vessel wall or to each other. However, when stimulated, they change shape and adhere to the vessel wall and other platelets and participate in hemostasis. Forming a platelet plug, they are instrumental in the stoppage of bleeding.

Humans possess an inbuilt system by which the blood normally remains in a fluid state and guards against the hazards of thrombosis and hemorrhage, and the mechanisms include:

1. The rapid flow of blood, which keeps the clotting factors at lower concentrations.
2. Anti-thrombin III, which inactivates all the clotting factors in the blood as well as any thrombin formed in circulation.
3. Removal of traces of fibrin formed in circulation by fibrinolysis.

## ANTI-PLATELET THERAPY

Known in lay terms as ‘Blood thinners,’ they are a group of drugs that decrease platelet aggregation and inhibit thrombus formation. They are effective in the arterial circulation, where anticoagulants have little effect.

## CLASSIFICATION OF ANTI-PLATELET AGENTS

Till recently, aspirin was the only drug available, however, of late, several additional drugs have been added. The availability of several such drugs has given a wider choice to the clinician to consider each situation with a different approach [Tables 1 and 2].

**Table 1:** Based on mechanism of action

Platelet anti-activators	Platelet anti-aggregants
Aspirin	Antagonists of GP IIb/IIIa
Flurbiprofen	receptor
Dipyramidol	

**Table 2:** Based on the site of action

Site of action	Anti-platelet agent
Cyclooxygenase inhibitors	Aspirin
Adenosine diphosphate receptor inhibitors	Clopidogrel, ticlopidine
Phosphodiesterase inhibitors	Cilostazol
Glycoprotein IIb/IIIa inhibitors	Abciximab, eptifibatide, tirofiban, defibrotide
Adenosine reuptake inhibitors	Dipyridamole

## ASPIRIN

Developed in 1897, aspirin is one of the world's safest and least expensive drugs, with proven effects for over 100 years. It has been widely prescribed for various ailments, but by far it is most used for relief from pain, especially in patients with arthritis.

Lawrence Craven, a suburban general practitioner in Glendale, California, reported that daily, low doses of aspirin could prevent myocardial infarction and stroke. None of his 400 patients who received aspirin for a two-year period (1948–1950) developed myocardial infarction.[2] This was probably the first attempt to prescribe and rationally use aspirin to prevent myocardial infarction.

Mechanism of action: Irreversibly inhibits the enzyme cycloxygenase (COX), resulting in reduced platelet production of TXA_2_ (potent vasoconstrictor and platelet aggregant).[3]

**Dose and administration:** At least 160 mg soluble aspirin is required to maximally inhibit platelet function within 30 minutes. Daily doses accumulate and it has been seen that doses as low as 40 mg/day are effective. A loading dose as high as 300 mg is sometimes recommended when an immediate antithrombotic effect is required. The present consensus states that a daily aspirin dosage of 75 to 150 mg is recommended for the long-term prevention of serious vascular events in high risk patients^4^. Increasing to higher doses does not necessarily improve platelet inhibition and in fact, may be counterproductive.

## ANTI-PLATELET DRUGS AVAILABLE IN INDIA

One needs to be familiar with the anti-platelet drugs available in India and the various formulations, dosage and side effects are included in the table [Table 3].

**Table 3:** Anti-platelet drugs available in India

Name	Dose loading preventive	Complications
Aspirin	300 mg 75-150 mg	GI bleeding, urticaria, tinnitus
Clopidogrel	300-600 mg 75 mg	GI bleeding, thrombocytopenia, diarrhoea
Ticlopididine	250 mg twice daily	Thrombocytopenia, neutropenia, skin rash, diarrhoea
Tirofiban	Bolus - 0.4-0.6 mg/kg Infusion - 0.10-0.15 μg/kg/min	-

Oral anti-platelet regimens: Vary in different institutions and to quote a recommendation from the American College of Chest Physicians 2006.[1]

Low-dose aspirin remains the cornerstone of oral anti-platelet therapy. Current recommendations for this situation are:

- For all patients with chronic stable coronary artery disease (CAD), aspirin should be given (75–162 mg) and continued indefinitely.
- For all patients with stable CAD, with a risk profile indicating a high likelihood of developing acute myocardial infarction (AMI), long-term clopidogrel in addition to aspirin is given.

Dual oral anti-platelet therapy was introduced relatively recently. When used with aspirin, it provides an additional 20% reduction in the relative risk of MI or stroke, as compared to aspirin alone.

Dual therapy with aspirin and clopidogrel is recommended in cases of non ST-elevation, acute coronary syndrome (ACS), and ST-elevation MI. In some situations both aspirin and clopidogrel are started and clopidogrel withdrawn after 9 to 12 months.

## BLEEDING VERSUS THROMBOEMBOLIC COMPLICATIONS

Surgical procedures and resultant bleeding is a normal event, but in the presence of anti-platelet therapy, it assumes clinical significance and poses a dilemma to the clinician. The question is whether to stop therapy before the procedure and avoid possible excessive bleeding or discontinue therapy and attract serious thromboembolic events.

Traditionally it was recommended to discontinue aspirin use for 7 to 10 days or at least for three days. However, scientific evidence shows that interruption of oral anti-platelet therapy is associated with a progressive recovery of platelet function and with a potential rebound of thrombotic arterial events. Pro-thrombotic effects overcome the physiological balance. Excessive thromboxane A_2_ activity and decreased fibrinolysis have been noted on stopping aspirin. Therefore, a benefit–harm relationship should be established, keeping in mind the systemic health and other predisposing factors.[1]

Four hundred and seventy-four studies reviewed by Burger *et al*.,[5] on the impact of low-dose aspirin on surgical blood loss, showed that patients on aspirin alone have an average intraoperative hemorrhagic risk increased by a factor of 1.5, without an increase in surgical mortality or morbidity.

A systematic review and meta-analysis on the hazards of discontinuing aspirin further confirms the major detrimental impact and ominous prognostic implication of withdrawal across a large spectrum of subjects (50,279) at risk for *de novo* or recurrent cardiovascular events.[6]

A case of proximal deep vein thrombosis was reported after a sudden stoppage of clopidogrel suspension.[7] In one of the largest meta-analysis, namely, Anti-platelet Trialist's collaboration, involving 70,000 subjects, it was reported that long-term anti-platelet therapy caused a reduction of mortality by 10%, 31% reduction in the relative risk of occurrence of ischemic myocardial attack (IMA), and 18% reduction in the relative risk of occurrence of ischemic cerebrovascular attack (ICA), associated with atherosclerosis. Spontaneous hemorrhage on the other hand, increased by 0.12% only.[8] The inference from this would be that those undergoing surgery, who interrupted anti-platelet therapy, exposed themselves to a higher risk of recurrence of thrombosis.

## BLEEDING AFTER INVASIVE DENTAL PROCEDURES IN PATIENTS UNDER ANTI-PLATELET THERAPY

Postoperative hemorrhagic complications can be severe and may require aggressive interventions including hospitalization. Minor hemorrhages are more common and dealt with by routine office procedures.

A literature review and guideline development process conducted by the Oral Medicine and Oral Surgery Francophone Society found that, based on the agreement among professionals in the field, interruption of therapy before dental procedures is unnecessary. Many similar procedures carry a low risk of bleeding, and any bleeding that occurs can usually be controlled by local hemostasis.[9]

Other than invasive procedures it also has an impact on clinical assessments such as bleeding on probing. In one trial, 54 patients were divided into three groups. The first group took 81 mg aspirin for seven days, a second group took 325 mg aspirin daily for seven days, and the third group took placebo daily for the same duration. This study concluded that the effects of aspirin could impair diagnostic assessments and treatment planning decisions for the clinicians.[10]

In a prospective study by Ardekian *et al*.[11] 39 patients taking aspirin were studied. Nineteen continued the anti-platelet therapy, while 20 stopped taking aspirin seven days prior to the extractions. Intraoperative bleeding was controlled in 33 patients with gauze packs and sutures. Six patients had tranexamic acid added to the local packing. Finally, it was observed that no patient experienced bleeding immediately or in the week following the procedure.

In a retrospective study of 43 patients on single or dual anti-platelet therapy who underwent 88 invasive procedures consisting of extractions, periodontal surgery, and subgingival scaling and root planing, Napenas *et al*.[12] found no differences between patients receiving single or dual anti-platelet therapy.

A prospective observational study was used to quantitatively assess the amount and severity of bleeding encountered with dentoalveolar surgery in two groups, one on anti-platelet therapy, and the other, a group of healthy controls. They demonstrated no difference in blood loss after a minor oral surgical procedure.[13]

A prospective trial on 155 patients under anti-platelet therapy reaffirms the fact that local measures are sufficient to control post-extraction hemorrhages. It seems advisable to be cautious with regard to the number of teeth to be extracted during the same session, and it has been recommended that not more than three teeth are to be extracted at a time, and that these should either be adjacent or correlative, and not in different parts of the dental arch. For molar teeth, no more than two adjacent teeth should be extracted.[14]

There is controversy among dentists and physicians regarding the appropriate dental management of patients receiving dual anti-platelet therapy, due to the lack of clinical studies about hemorrhagic risk in these patients. Options before a dental clinician includes modifying dual anti-platelet therapy by altering the dosage or switching to monotherapy or discontinuing therapy.

However, when a definite increase in intraoperative bleeding is feared, or when surgical hemostasis is difficult, aspirin can be replaced by a shorter acting nonsteroidal anti-inflammatory drug, given for a 10-day period and interrupted the day before surgery, and postoperative anti-platelet treatment should be resumed immediately after surgery (first six hours).[4]

A recent consensus opinion from the American Heart Association, American College of Cardiology, Society for Cardiovascular Angiography and Interventions, American College of Surgeons, and American Dental Association recommended continuing aspirin and clopidogrel therapy for minor dental surgical procedures in patients who have coronary artery stents, or delaying treatment until the prescribed regimen is completed.[15]

The hemorrhagic risk related to dental extraction is a rare complication. The incidence of post-extraction hemorrhagic complications, including other risk factors, does not exceed the average of 0.2 and 2.3%.[16]

In specific extenuating circumstances, if discontinuation is essential, it should be limited to three or fewer days, as increased risk for thrombotic events increases when discontinuation is between four and 30 days.[17]

## ANTI-PLATELET THERAPY AND ISOLATED REPORTS OF SEVERE HEMORRHAGE

Anti-platelet therapy is exposed to only a minor risk of hemorrhagic attack. Literature reveals few case reports of severe hemorrhage associated with the intake of aspirin.

McGaul *et al.*[18] reported a case of sublingual hematoma, following a periodontal flap procedure in the region of the lower anterior teeth, in a patient on long-term aspirin therapy. The hematoma resolved on its own, but systemic antibiotics was given to control infection.

Thomason[19] reported severe bleeding following gingivectomy in a patient receiving 150 mg of aspirin, which later needed platelet transfusion. However, the patient was a renal transplant recipient and it was difficult to precisely establish the immutability of aspirin for such an incident, keeping in mind the other numerous hemorrhagic risk factors.

Scher[20] advocated stopping aspirin before any surgical procedure performed on a non-emergency basis. He found that diffuse postoperative bleeding was associated with preoperative use of aspirin. Patients in this study, however, were receiving higher dosages of aspirin.

Elad *et al*.[21] reported a case of critically severe bleeding following non-surgical periodontal treatment in a patient whose platelet count and bleeding time were normal, with INR at 1.

## PATIENTS ON HIGHER DOSAGES OF ASPIRIN

Often, patients are on high dosages of aspirin (more than 500 mgs) for pain relief and the therapeutic goal is not the prevention of thromboembolic events. Aspirin therapy can be discontinued in such patients without attracting any adverse thromboembolic events. Possible excessive bleeding, however, has to be anticipated. Literature reports one case of severe, hardly controllable hemorrhage in a male patient of 62 years after extraction of his teeth. Platelet transfusion was necessary to stop the bleeding.[22]

## THINGS TO KEEP IN MIND WHILE MANAGING PATIENTS UNDER ANTI-PLATELET THERAPY FOR DENTAL CURE

The main biological tests capable of evaluating the repercussion of the anti-platelet therapy on hemostasis are: measurement of the bleeding time (BT), PFA (platelet function analyzer), and analysis of the platelet functions by aggregametry or by flow cytometry. Other than the measurement of the BT, all the other biological tests require specialized laboratory tests and they cannot be used in a routine set up.[23]

According to Scully and Wolf,[24] oral surgical procedures must to be done at the beginning of the day, as it allows more time to deal with any bleeding problem that exists. Also procedures must be performed early in the week, which gives adequate time allowance for any late bleeding episodes on weekdays.

The continuation of anti-platelet treatment does not contraindicate the use of local anesthesia, but regional nerve blocks are to be avoided when possible. If no alternative exists local anesthesia can be deposited with caution and continuous aspiration.

Resorbable sutures are preferred. After closing, pressure is applied to the socket using a gauze pad and the patient is asked to bite on that for 15 to 30 minutes. All efforts should be made to make the procedure as atraumatic as possible.

Most of the time, if the bleeding persists; it is controlled and managed with the use of local hemostatic agents. It is recommended to give the patient a written copy of postoperative instructions to follow, in case of bleeding. Use of mouthwashes containing tranexamic acids is also recommended.

Use of aspirin leads to significant bleeding time. if the bleeding time exceeds greater than 20 minutes and surgery has to be performed as an emergency procedure, 1-desamino-8-D-arginine vasopressin (DDAVP) can be used to shorten the bleeding time.[25]

The mechanism of action is not clear, but it involves enhancement of the von Willebrand's factor, which acts as a platelet aggregant. DDAVP can be administered parenterally at a dose of 0.3 μg/kg of body weight not exceeding 20 – 24 μg or as a nasal spray.

Use of DDAVP should be done in consultation with the patient's physician or hematologist. It should be used with caution in older patients because of the potential risk of causing drug- induced thrombosis.

## CONCLUSION

Anti-platelet drugs are increasingly being used and a greater number of such patients are presenting for dental treatment. Clinicians are confronted with the dilemma of either stopping the drugs and inviting a remote but possible adverse thromboembolic event, or continuing the drugs and facing the eventuality of excessive bleeding from minor dental procedures.

Evidence collated from sound scientific sources advocates continuation of drugs, and a clinician may be treading on safer turf if he/she adheres to such advice. Looking at the other side of the coin is the prospect of more than usual bleeding from dental procedures. While it is easily said that such bleeding is ably controlled, sporadic and frightening reports are likely to deter the clinician from undertaking invasive dental procedures, while continuing anti-platelet therapy.

The risk of bleeding is not homogeneous across all patient groups and bleeding with continued anti-platelet therapy could be coincidental.

There are also legal considerations to ponder about, but either way a clinician can always defend his actions.

The by word would be caution, prudence and base one's judgment, weigh the evidence, and proceed on a case-by-case basis.

Information so far available on the risk of anti-platelet discontinuation or continuation and the adverse outcomes, rely on the retrospective studies or meta-analyses of small observational studies. Conclusive evidence from large interventional studies should be able to throw more light on this aspect.

Newer PAR-1 antagonists under trial are likely to clear the dilemma.. They are promising effective anti-platelet action without any associated significant bleeding.[26]

While we wait for new evidence to emerge, it is safe to accept the view that continuation of anti-platelet therapy has minimal impact on the amount and nature of post event bleeding, and the consequences of thromboembolic events far outweigh the risk of bleeding from dental procedures.
